# Incomplete male puberty: a new nosographic entity

**DOI:** 10.1007/s12020-026-04732-2

**Published:** 2026-08-08

**Authors:** Sandro La Vignera, Rosita A. Condorelli

**Affiliations:** Department of Clinical and Experimental Medicine, University of Catania, Catania, Italy

**Keywords:** Incomplete male puberty, Sertoli cell dysfunction, AMH, Inhibin B, Minipuberty, Cryptorchidism, Endocrine disruptors, Testicular volume, Male infertility

## Abstract

**Purpose:**

Incomplete male puberty is a recently proposed nosographic entity defined by the development of normal secondary sexual characteristics in boys who nonetheless fail to achieve adequate testicular volume (<12 mL) during puberty due to underlying Sertoli cell dysfunction. The condition typically goes unrecognised until the first spermiogram reveals azoospermia or severe oligozoospermia. This review examines the pathophysiology, available diagnostic tools, and clinical management strategies.

**Methods:**

A comprehensive literature search was conducted across SciSpace, PubMed, and Google Scholar covering Sertoli cell biology, AMH and Inhibin B as biomarkers, minipuberty physiology, endocrine disruptors, cryptorchidism, maternal metabolic disorders, and nutritional influences on testicular development. More than 500 relevant publications were retrieved and synthesised. This is a narrative review; no standardized or systematic methodology was applied for manuscript selection.

**Results:**

Minipuberty (months 1–6 of life) represents the earliest and most informative diagnostic window: Inhibin B peaks at 378 ± 23 pg/mL and AMH rises sharply, both reflecting Sertoli cell mass and function. Inhibin B below 60 pg/mL during this window predicts poor spermatogenic outcomes. Pubertal testicular volume progression below the P10 centile, or failure to reach ≥12 mL in adulthood, constitutes a cardinal sign. High-risk categories include cryptorchidism (bilateral Sertoli impairment in 70% of unilateral cases), offspring of diabetic or hypothyroid mothers, and boys exposed to endocrine-disrupting chemicals in utero.

**Conclusion:**

A structured surveillance protocol combining early infancy hormonal screening during minipuberty, serial orchidometry throughout childhood, and targeted Sertoli cell stimulation tests can identify incomplete male puberty years before irreversible spermatogenic failure. Early recognition enables timely intervention and counselling.

## Introduction

Male puberty is a complex neuroendocrine process orchestrated by the reactivation of the hypothalamic–pituitary–gonadal (HPG) axis after the juvenile quiescent period. The hallmark of pubertal testicular maturation is a progressive increase in testicular volume driven by Sertoli cell proliferation and the expansion of the seminiferous tubular compartment. Testicular volume is therefore a reliable proxy of Sertoli cell mass and, ultimately, of the future spermatogenic capacity of the individual [1, 2].

When assessing testicular volume development against age-based centile charts, bone age rather than chronological age should be preferred, particularly in boys with constitutional delay of growth and puberty, in whom chronological age alone would overestimate the degree of testicular underdevelopment.

Clinicians have long recognised conditions of frank hypogonadism—Klinefelter syndrome, congenital hypogonadotropic hypogonadism (CHH), and post-orchitis atrophy—in which both secondary sexual characteristics and testicular growth are impaired. However, a distinct and hitherto poorly characterised phenotype exists in which boys progress through puberty with apparently normal virilisation yet harbour testicles of inadequate size. This entity, which we propose to term incomplete male puberty, results from Sertoli cell dysfunction that escapes clinical detection during adolescence and manifests only when the first spermiogram reveals azoospermia or severe oligozoospermia [3, 4]. It is important to recognise that established conditions—most notably Klinefelter syndrome during adolescence and Sertoli/seminiferous tubular damage secondary to chemotherapy or radiotherapy—may present with an indistinguishable clinical picture (normal virilisation, small testes, low Inhibin B and AMH, subsequent azoospermia) for several years, and must therefore be excluded by appropriate investigation before this diagnosis is applied (F1).

The present review addresses the following key questions: (1) What tools are available to detect incomplete male puberty at the earliest possible stage? (2) Which parameters best assess the correct maturation of testicular volume during puberty? (3) Do hormonal tests—particularly AMH and Inhibin B—demonstrate Sertoli cell function adequately in the pre-pubertal phase? (4) Should Sertoli cell stimulation tests be proposed routinely in the pre-pubertal period? (5) Should knowledge of minipuberty be improved and early infancy screening in high-risk populations proposed [universal screening is not currently supported by high-quality evidence and raises significant practical and cost–benefit considerations]? (6) How do metabolic factors and nutritional status affect the pre-pubertal testis? (7) How do endocrine disruptors affect fetal testicular programming? (8) Which high-risk categories merit enhanced surveillance?

## Sertoli cell biology and the pathophysiology of incomplete male puberty

### Sertoli cell proliferation and maturation

Sertoli cells are the only somatic cells of the seminiferous epithelium and serve as the primary determinants of spermatogenic capacity. Their total number, established during two critical proliferative windows—fetal life and early postnatal minipuberty (months 1–6)—sets a ceiling on adult sperm production that cannot be exceeded [5]. Each mature Sertoli cell supports a finite number of germ cells; therefore, a reduced Sertoli cell complement inevitably limits spermatogenic output.

Sertoli cell maturation is marked by the loss of proliferative capacity and the acquisition of a fully differentiated secretory phenotype, characterised by the expression of androgen receptor (AR), tight junctions forming the blood–testis barrier, and the downregulation of AMH production. This transition is driven by rising intratesticular testosterone and FSH signalling at puberty. When Sertoli cell maturation is incomplete—due to genetic, environmental, or nutritional insults—the seminiferous tubules remain underdeveloped, testicular volume fails to reach the normal adult range, and spermatogenesis is profoundly impaired [6].

### AMH and Inhibin B as sertoli cell biomarkers

Anti-Müllerian hormone (AMH) is produced exclusively by Sertoli cells from fetal life until puberty. In the neonatal period, AMH concentrations rise sharply during minipuberty and remain elevated throughout childhood, reflecting the immature, proliferating Sertoli cell phenotype. At puberty, rising testosterone suppresses AMH to low adult levels. Inhibin B, a heterodimeric glycoprotein secreted by Sertoli cells in response to FSH, provides a complementary measure of Sertoli cell function and germ cell co-stimulation [7, 8].

During minipuberty, Inhibin B peaks at 378 ± 23 pg/mL at approximately 3 months of age, reflecting maximal Sertoli cell activity. Values below 60 pg/mL during this window have been associated with poor long-term spermatogenic outcomes (Table 1). The LH/FSH ratio greater than 0.32 during minipuberty separates boys at risk for CHH from healthy controls with a positive predictive value of 93% [9].

**Table 1:** AMH and Inhibin B reference ranges by developmental stage

Stage	Age	AMH (pmol/L)	Inhibin B (pg/mL)
Minipuberty	1–3 months	250–750	200–500 (peak ~ 378 ± 23)
Late minipuberty	3–6 months	200–600	150–400
Pre-pubertal childhood	6 months – 8 years	100–500	50–200
Early puberty (Tanner G2–G3)	9–12 years	50–200	80–250
Late puberty (Tanner G4–G5)	13–17 years	5–50	100–350
Adult	>18 years	2–20	80–300
Incomplete male puberty (adult)	>18 years	Variable	<60 (associated with azoospermia)

Note: AMH and Inhibin B decline physiologically from late puberty onwards; low values in adulthood are therefore not inherently pathological and must be interpreted in the context of spermiogram results

## Testicular volume as a clinical parameter

### Orchidometry and ultrasound

Testicular volume is the most accessible and informative clinical measure of pubertal testicular maturation. The Prader orchidometer remains the standard clinical tool, although scrotal ultrasonography with three-dimensional volume calculation (length × width × height × 0.71) provides greater precision. Pubertal onset is conventionally defined as testicular volume ≥ 4 mL (Tanner G2), occurring at a median age of 11.5 years in healthy-weight boys [10].

Serial orchidometry during puberty is essential for identifying boys with incomplete testicular growth. A testicular volume below the P10 centile for chronological age, or failure to achieve ≥ 12 mL in adulthood, should prompt hormonal evaluation. The normal adult range is 15–25 mL (mean 18–20 mL). In incomplete male puberty, testicular volume plateaus in the 6–10 mL range despite normal penile growth, pubic hair, and axillary hair development.

### Centile charts and population norms

**Fig. 1:**
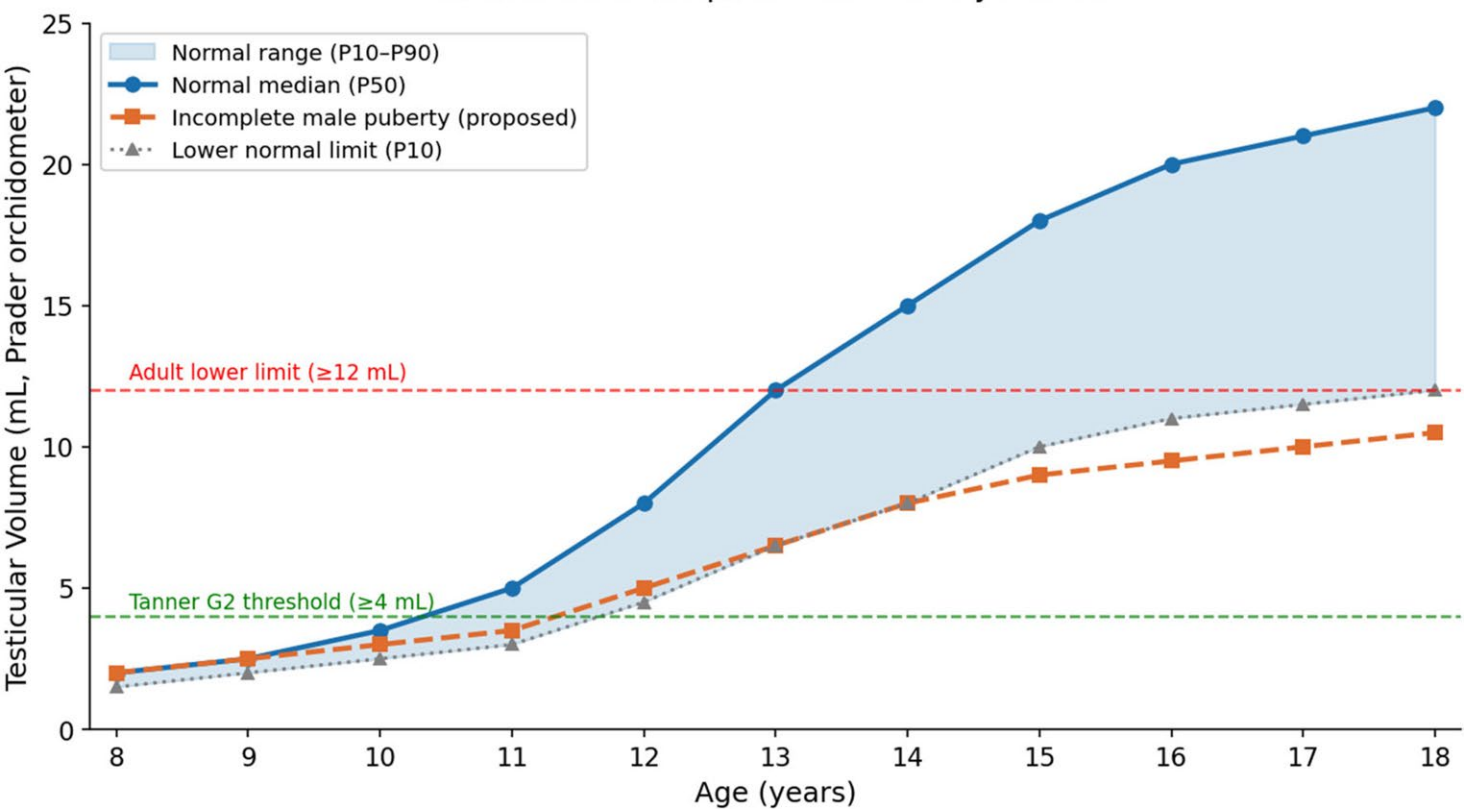
Testicular volume progression during male puberty. The shaded area represents the P10–P90 range in healthy boys. Boys with incomplete male puberty (red dashed line) show a characteristic plateau below the lower normal limit (≥12 mL) despite normal secondary sexual characteristics. Tanner G2 onset threshold is indicated at 4 mL

Population-based centile charts for testicular volume from birth to adulthood are an underutilised tool in paediatric endocrinology. Cross-sectional data from European and North American cohorts provide P10, P50, and P90 reference ranges that can be used to identify boys whose testicular growth trajectory falls below expected norms. Longitudinal tracking—analogous to height velocity charts—would substantially improve early detection of incomplete male puberty [11] (Fig. 1).

### Hormonal correlates of testicular volume

**Fig. 2:**
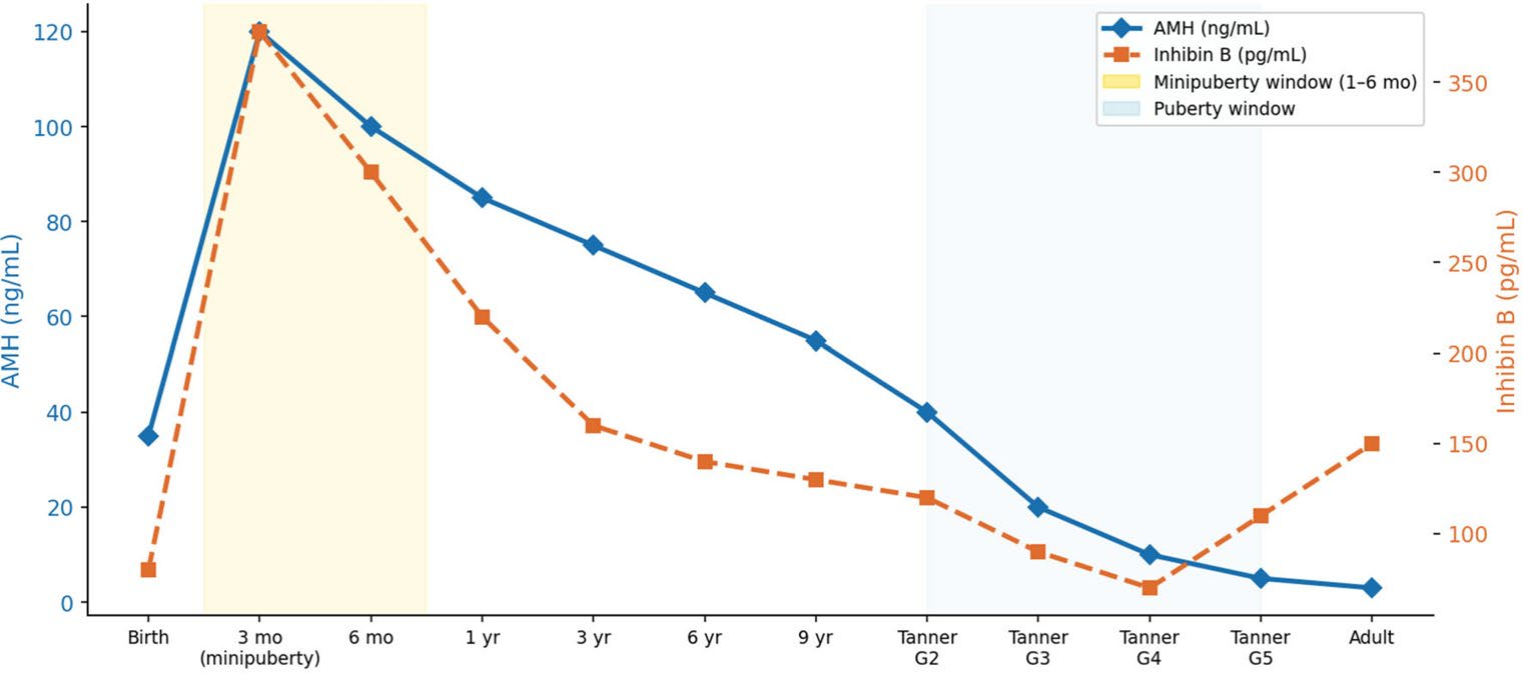
AMH and Inhibin B serum trajectories from minipuberty to adulthood as markers of Sertoli cell function. The gold shaded window (1–6 months) represents the minipuberty period. Inhibin B peaks at 378 ± 23 pg/mL at 3 months. AMH remains elevated throughout childhood and declines at puberty

Testicular volume correlates strongly with Sertoli cell markers throughout development. Inhibin B and AMH trajectories from minipuberty to adulthood mirror the expansion and subsequent maturation of the Sertoli cell pool (Fig. 2). Boys with incomplete male puberty show a blunted Inhibin B rise during minipuberty and a failure to achieve the expected Inhibin B plateau during mid-childhood, preceding the detectable deficit in testicular volume by several years.

## Minipuberty: the earliest diagnostic window

### Physiology of minipuberty

Minipuberty refers to the transient activation of the HPG axis that occurs in the first 3–6 months of postnatal life. In male infants, this surge in LH and FSH stimulates Leydig cell testosterone production and Sertoli cell proliferation, establishing the Sertoli cell complement that will determine adult spermatogenic capacity. Testosterone peaks at 1–3 months (median 250 ng/dL), and Inhibin B peaks at 3 months (378 ± 23 pg/mL) [12]. Although the hormonal peak in males is reached at approximately 3 months of age, minipuberty may extend up to 6 months in males and up to 24 months in females.

The biological significance of minipuberty extends beyond gonadal activation: it also programmes brain masculinisation, penile growth, and the final descent of the testes. [The evidence for minipuberty-driven brain masculinisation in humans remains limited; this claim warrants further dedicated references and may be controversial.] Boys with CHH may already exhibit small testes at birth, as poor testicular development begins during fetal life. Furthermore, those who lack minipuberty have smaller testes, smaller penile length, and lower Inhibin B at diagnosis compared with boys who experienced a normal minipubertal surge, underscoring the long-term consequences of this neonatal window [13].

### Minipuberty screening: rationale and protocol

Given the critical importance of minipuberty for Sertoli cell establishment, early infancy screening of AMH and Inhibin B at 1–3 months of age could identify boys destined for incomplete male puberty years before clinical manifestations. A proposed screening panel would include: serum LH, FSH, testosterone, AMH, and Inhibin B at 1 and 3 months; testicular ultrasound at 3 months; and repeat hormonal assessment at 6 months if initial values are borderline [14] (Table 2).

**Table 2:** Minipuberty screening panel and interpretation guide

Parameter	Timing	Normal range (1–3 months)	Action if abnormal
LH	1 and 3 months	0.5–4.0 IU/L	If <0.5: evaluate for CHH; if >4.0: evaluate for primary hypogonadism
FSH	1 and 3 months	0.5–6.0 IU/L	If elevated (>10 IU/L): suggests primary testicular failure
Testosterone	1–3 months	50–400 ng/dL	If <50: evaluate Leydig cell function; hCG stimulation test
AMH	1–3 months	250–750 pmol/L	If <100: reduced Sertoli cell mass; enhanced surveillance
Inhibin B	3 months	200–500 pg/mL	If <60: high risk for spermatogenic failure; FSH stimulation test at age 6–8 years
LH/FSH ratio	3 months	<0.32	If >0.32: 93% PPV for CHH; refer to paediatric endocrinology
Testicular position (US)	3 months	>0.5 mL each	If <0.3 mL: evaluate for cryptorchidism or testicular dysgenesis

Widespread neonatal minipuberty screening is not currently standard practice in any country. The evidence base for its clinical utility is growing but remains limited to retrospective cohort studies and case series. A prospective multicentre trial assessing the predictive value of minipuberty hormones for adult spermatogenic function is urgently needed.

## Pre-pubertal hormonal assessment and sertoli cell stimulation tests

### Basal hormonal profile

In the pre-pubertal boy (ages 6 months to 9 years), the HPG axis is quiescent and basal LH and FSH are low. Despite this, AMH remains measurable and high (reflecting immature Sertoli cells), and Inhibin B is detectable at levels that correlate with Sertoli cell number. A pre-pubertal Inhibin B below the age-specific P5 centile, or an AMH below the P10 centile, should raise concern about reduced Sertoli cell mass [15].

### hCG and FSH stimulation tests

The human chorionic gonadotrophin (hCG) stimulation test assesses Leydig cell reserve and has been used for decades to diagnose anorchia and evaluate cryptorchidism. More recently, FSH stimulation has been proposed as a means of evaluating Sertoli cell responsiveness in the pre-pubertal period: an adequate Inhibin B rise after recombinant FSH administration confirms functional Sertoli cells, while a blunted response suggests impaired Sertoli cell mass or function [16].

The clinical utility of pre-pubertal Sertoli cell stimulation tests has not been established in large prospective studies. Current evidence suggests that an Inhibin B increment of less than 30 pg/mL after FSH stimulation is associated with a significantly higher risk of spermatogenic failure in adulthood. Standardisation of the stimulation protocol (dose, timing, reference values) is needed before widespread adoption.

## Environmental and endocrine disruptors

### Fetal programming of testicular function

The testicular dysgenesis syndrome (TDS) hypothesis proposes that cryptorchidism, hypospadias, impaired spermatogenesis, and testicular germ cell tumours share a common origin in disrupted fetal testicular programming during the masculinisation programming window (weeks 8–14 of gestation). Endocrine-disrupting chemicals (EDCs) that interfere with androgen signalling during this critical window can permanently reduce Sertoli cell number and impair testicular descent [17].

### Phthalates

Dibutyl phthalate (DBP) and di-2-ethylhexyl phthalate (DEHP) are the most extensively studied reproductive toxicants. In rodent models, gestational exposure to DBP reduces fetal testosterone production, causes cryptorchidism and hypospadias, and permanently reduces Sertoli cell number. Epidemiological studies in humans have linked urinary phthalate metabolites during pregnancy to reduced anogenital distance (AGD) and smaller testicular volume in male offspring [17, 18].

### Atrazine and pesticides

Atrazine, one of the most widely used herbicides globally, induces cryptorchidism, hypospadias, and reduced AGD in rodents through downregulation of INSL3 (insulin-like factor 3), a key mediator of testicular descent. Organochlorine pesticides (DDT, DDE) and polychlorinated biphenyls (PCBs) disrupt thyroid hormone signalling and neuroendocrine function, with downstream effects on Sertoli cell maturation [19].

### Hexavalent chromium and heavy metals

**Fig. 3:**
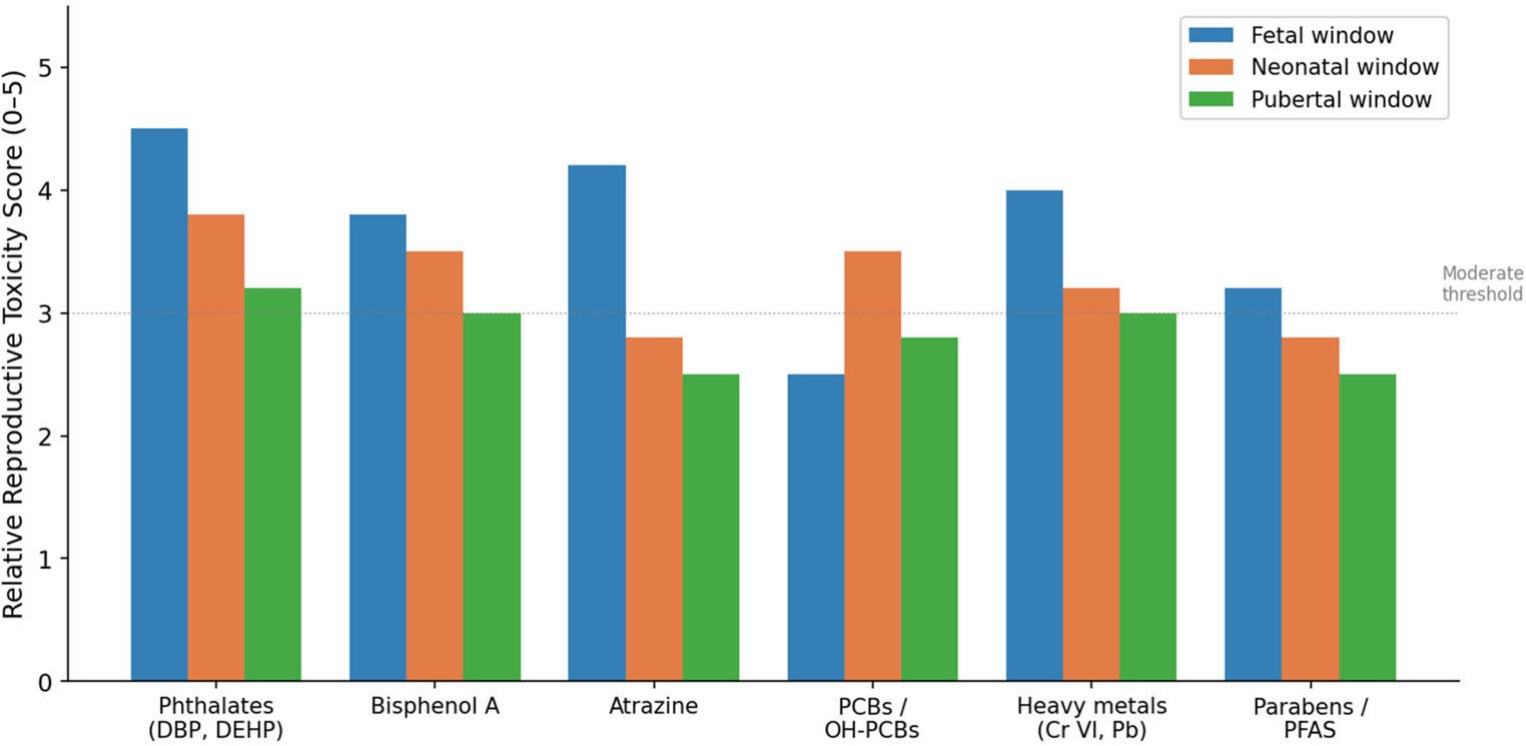
Relative reproductive toxicity of major endocrine-disrupting chemicals (EDCs) by developmental window. Phthalates and hexavalent chromium show the highest fetal toxicity scores. Toxicity scores are derived from published animal and epidemiological data

Hexavalent chromium (Cr VI) reduces fetal testosterone production, decreases Leydig cell number, and increases gonocyte (prospermatogonial) counts in exposed offspring, suggesting a shift toward an immature testicular phenotype. Lead and cadmium have been associated with reduced Inhibin B and increased FSH in epidemiological studies of occupationally exposed adults [20] (Table 3; Fig. 3).

**Table 3:** Major endocrine-disrupting chemicals and reproductive effects

EDC class	Key compounds	Mechanism	Male reproductive effect
Phthalates	DBP, DEHP	Anti-androgenic; inhibit fetal testosterone synthesis	Cryptorchidism, hypospadias, reduced AGD, reduced Sertoli cell number
Herbicides	Atrazine	INSL3 downregulation; aromatase induction	Cryptorchidism, hypospadias, reduced AGD
Organochlorines	DDT, DDE, PCBs	Thyroid disruption; oestrogen mimicry	Reduced Inhibin B, impaired Sertoli maturation
Heavy metals	Cr(VI), Pb, Cd	Oxidative stress; Leydig cell toxicity	Reduced testosterone, reduced Inhibin B, gonocyte accumulation
Bisphenols	BPA, BPS	Oestrogen receptor agonism; AR antagonism	Reduced sperm quality, altered HPG axis programming
Parabens	Methylparaben, propylparaben	Weak oestrogen agonism	Reduced sperm motility; potential Sertoli effects

## Metabolic factors and nutrition

### Obesity and early pubertal onset

Childhood obesity is associated with earlier pubertal onset (median 11.3 years in obese boys vs. 11.5 years in healthy-weight boys) through leptin-mediated activation of GnRH neurones. Despite earlier onset, obese boys tend to have reduced adult testicular volume and lower sperm production, possibly due to scrotal hyperthermia, oxidative stress, and adipokine-mediated suppression of the HPG axis [21].

### Caloric restriction and undernutrition

Animal studies consistently demonstrate that caloric restriction delays reproductive development, reduces testicular weight, and decreases sperm production. In boys, severe malnutrition during early childhood is associated with delayed puberty and reduced adult testicular volume. Conversely, enhanced nutritional status accelerates Sertoli cell maturation and increases testicular size, suggesting that nutritional sufficiency is a prerequisite for optimal testicular programming [21].

### Insulin resistance and metabolic syndrome

Insulin resistance and metabolic syndrome are increasingly prevalent in adolescent boys and are associated with lower testosterone, higher oestradiol (via adipose aromatase activity), and reduced Inhibin B. These hormonal changes may contribute to incomplete Sertoli cell maturation and suboptimal testicular growth during puberty. Prospective studies examining the relationship between adolescent metabolic health and adult spermatogenic function are warranted.

## High-risk categories requiring enhanced surveillance

### Cryptorchidism

**Fig. 4:**
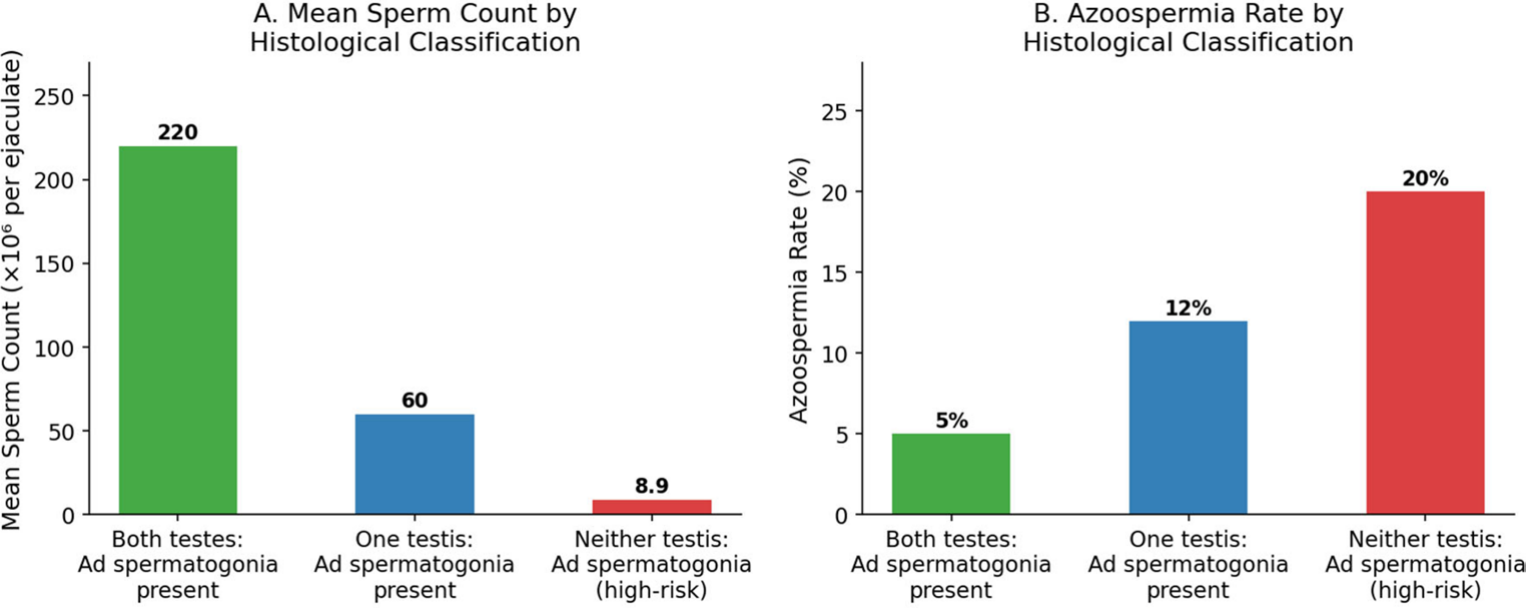
Fertility outcomes in post-pubertal men with a history of cryptorchidism, stratified by histological classification (presence or absence of Ad spermatogonia). Mean sperm count and azoospermia rates are shown for low-risk and high-risk groups

Cryptorchidism (undescended testis) is the most common congenital anomaly of the male genitalia, affecting 1–3% of term male infants. Even in unilateral cases, bilateral Sertoli cell impairment is demonstrable in 70% of patients, reflecting a systemic testicular dysgenesis rather than a purely mechanical problem. Boys with cryptorchidism and absent Ad spermatogonia at orchidopexy have a mean sperm count of only 8.9 × 10⁶/ejaculate in adulthood, compared with 220 × 10⁶/ejaculate in the low-risk group, and a 20% rate of azoospermia [3, 22] (Table 4; Fig. 4).

**Table 4:** Testicular histology in cryptorchidism and fertility outcomes

Histological group	Ad spermatogonia	Mean sperm count (×10⁶/ejaculate)	Azoospermia rate (%)
Low risk	Present (≥0.1/tubule)	220	<5
Intermediate risk	Reduced (<0.1/tubule)	45	10
High risk	Absent	8.9	20
Bilateral cryptorchidism	Variable	<20	30–40

Orchidopexy before 12–18 months of age is recommended to preserve germ cell number, but does not normalise Sertoli cell function in boys with intrinsic testicular dysgenesis. These boys should be enrolled in long-term surveillance programmes with serial AMH, Inhibin B, and testicular volume measurements throughout childhood and puberty.

### Offspring of diabetic mothers

**Fig. 5:**
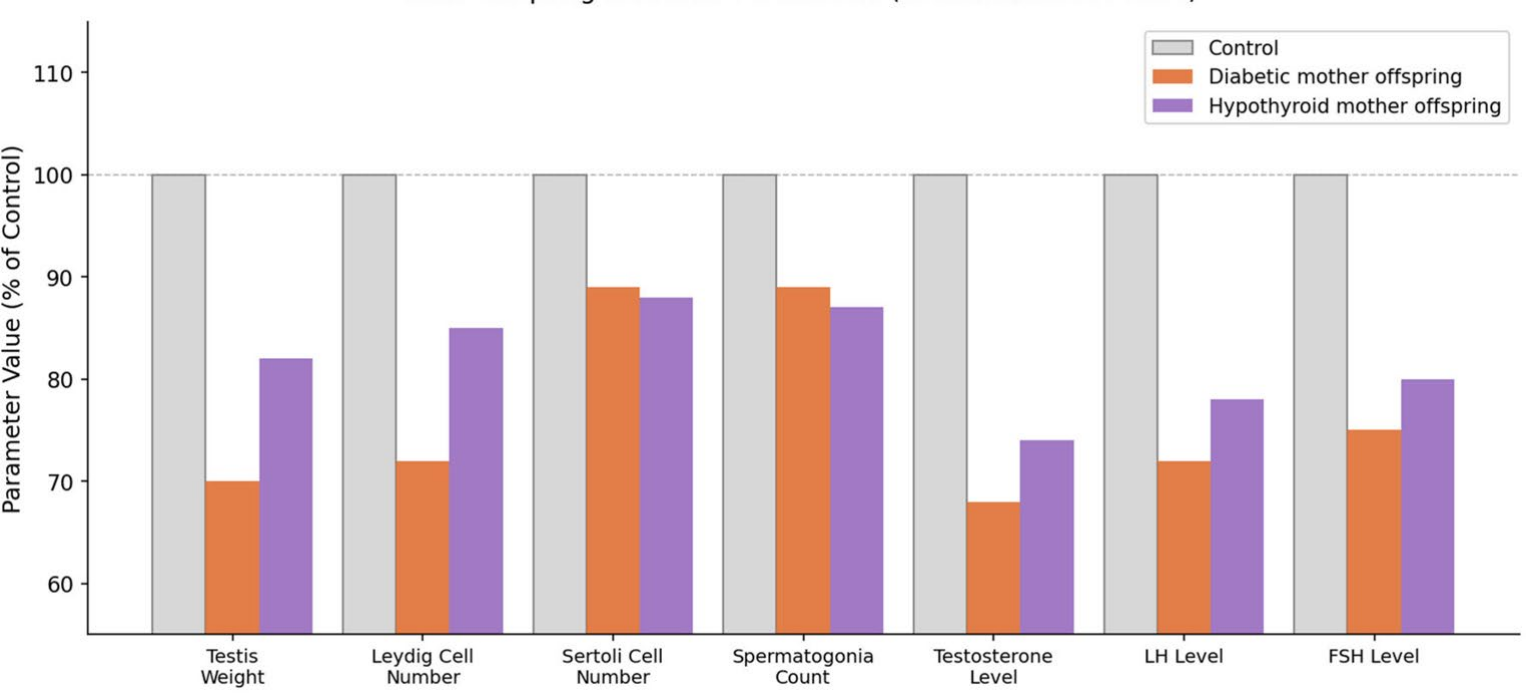
Effects of maternal diabetes and hypothyroidism on male offspring testicular parameters expressed as percentage of control. Maternal diabetes reduces testis weight (~ 30%), Leydig cell number (~28%), and Sertoli cell number (~11%)

Maternal diabetes mellitus—both pre-gestational and gestational—is associated with significant impairment of fetal testicular development. Animal models of streptozotocin-induced diabetes demonstrate a ~30% reduction in testis weight, ~28% fewer Leydig cells, and ~11% fewer Sertoli cells in male offspring (Fig. 5). The cord blood hCG–testosterone correlation (*r* = 0.64, *p* < 0.01) is disrupted in diabetic pregnancies, suggesting impaired Leydig cell responsiveness [23].

In human studies, boys born to mothers with gestational diabetes have lower testosterone and Inhibin B during minipuberty and smaller testicular volume at school age. These findings support the inclusion of male offspring of diabetic mothers in enhanced surveillance programmes from birth.

### Offspring of hypothyroid mothers

Maternal hypothyroidism during pregnancy is associated with reduced neonatal gonadotropin levels, lower testosterone in the first 6 months of life, smaller penile length, and reduced testicular volume in male offspring. Thyroid hormones are essential for Sertoli cell proliferation and maturation; their deficiency during critical developmental windows can permanently reduce Sertoli cell number. Adequate maternal thyroid hormone replacement prevents these effects, underscoring the importance of pre-conception and early gestational thyroid screening [24].

8.4 Boys with Genital Dimensions Below Normal Range Boys with micropenis (penile length > 2.5 SD below the mean for age) represent an additional at-risk category, as small genital dimensions may reflect underlying deficiencies in fetal testosterone secretion and, in some cases, may precede abnormalities in gonadotropin and testosterone secretion in later life. These boys should be included in the enhanced surveillance programme, with assessment of LH, FSH, testosterone, AMH, and Inhibin B from minipuberty onwards (Table 5).

**Table 5:** High-risk categories and recommended surveillance protocols

Risk category	Risk mechanism	Surveillance start	Recommended assessments
Cryptorchidism	Intrinsic testicular dysgenesis; germ cell loss	Birth / orchidopexy	Serial AMH, Inhibin B; testicular volume; spermiogram at 18 years
Maternal diabetes	Reduced fetal testosterone; Leydig/Sertoli cell deficit	Birth (minipuberty screen)	LH, FSH, testosterone, AMH, Inhibin B at 1–3 months; annual orchidometry
Maternal hypothyroidism	Thyroid hormone deficit → reduced Sertoli proliferation	Birth	Neonatal thyroid screen; minipuberty hormonal panel; annual orchidometry
EDC exposure in utero	Anti-androgenic programming; reduced Sertoli cell number [Note: EDC exposure in pregnancy is often difficult to document objectively; anamnestic data lack precision, and metabolite measurements require specialised laboratories. Inclusion in this algorithm therefore rests on epidemiological associations rather than direct causal proof.]	Birth	AGD measurement; minipuberty screen; annual orchidometry

## Proposed diagnostic algorithm

**Fig. 6:**
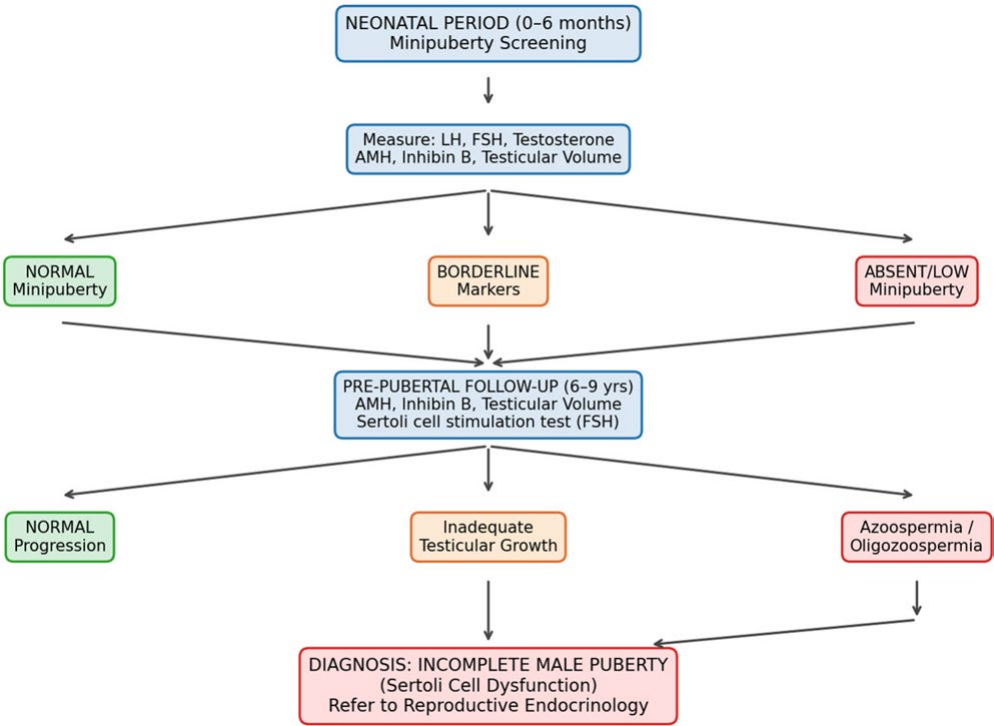
Proposed diagnostic algorithm for incomplete male puberty. The stepwise approach begins at neonatal risk stratification and proceeds through minipuberty assessment, pubertal monitoring, and post-pubertal confirmation. Colour coding indicates clinical outcome categories (normal, borderline, absent/low)

Based on the evidence reviewed, we propose a stepwise diagnostic algorithm for the early identification of boys at risk for incomplete male puberty (Fig. 6; Table 6). The algorithm begins at birth with risk stratification based on maternal history (diabetes, hypothyroidism, EDC exposure) and neonatal phenotype (cryptorchidism, hypospadias, reduced AGD). High-risk boys undergo minipuberty hormonal screening at 1–3 months; those with low Inhibin B (< 60 pg/mL) or absent minipubertal surge are classified as high risk and enter an enhanced surveillance pathway.

**Table 6:** Proposed diagnostic criteria for incomplete male puberty

Criterion	Definition	Clinical significance
Testicular volume	Adult volume < 12 mL bilaterally despite completed puberty	Cardinal sign; reflects Sertoli cell mass deficit
Pubertal growth plateau	Testicular volume < P10 for age during puberty	Early indicator; requires serial orchidometry
Sertoli cell markers	Inhibin B < 60 pg/mL at minipuberty or Inhibin B < 80 pg/mL at puberty	Functional Sertoli cell insufficiency
Spermiogram	Azoospermia or severe oligozoospermia (< 5 × 10⁶/mL) on first analysis	Confirms spermatogenic failure
Normal virilisation	Tanner G5, normal penile length, normal pubic/axillary hair	Distinguishes from frank hypogonadism
FSH plasma levels	Elevated FSH (hypergonadotropic) or low-to-normal FSH (hypogonadotropic) depending on level of dysfunction	Essential to differentiate primary gonadal damage from hypothalamic–pituitary dysfunction

During childhood (ages 2–9 years), annual orchidometry and biennial AMH/Inhibin B measurements are recommended for high-risk boys. At age 6–8 years, an FSH stimulation test may be considered to assess Sertoli cell reserve. At pubertal onset (age 9–11 years), testicular volume is tracked against centile charts; a volume below P10 for age triggers hormonal evaluation. Post-pubertally (age > 16 years), spermiogram analysis confirms or excludes spermatogenic failure.

## Clinical implications and future perspectives

The recognition of incomplete male puberty as a distinct nosographic entity has important implications for clinical practice. First, it shifts the diagnostic focus from adulthood (when spermatogenic failure is already established) to childhood and adolescence (when preventive and therapeutic interventions—particularly early orchidopexy for cryptorchidism—may still be effective; it must be acknowledged, however, that strong evidence for other therapeutic interventions to restore spermatogenesis in the pre-pubertal and adolescent period is currently lacking). Second, it identifies AMH and Inhibin B as clinically actionable biomarkers that should be incorporated into routine paediatric endocrinology practice for high-risk populations, using the following proposed thresholds: Inhibin B < 60 pg/mL during minipuberty or < 80 pg/mL at puberty, and AMH < 100 pmol/L during minipuberty, as indicators of Sertoli cell insufficiency warranting enhanced surveillance. Third, it highlights the importance of environmental, nutritional, and maternal factors in male reproductive programming.

Future research priorities include: (1) prospective multicentre studies validating minipuberty hormonal thresholds for predicting adult spermatogenic function; (2) randomised trials of early gonadotropin therapy in boys with absent or blunted minipuberty; (3) population-based centile charts for testicular volume from birth to adulthood; (4) standardised FSH stimulation test protocols for pre-pubertal Sertoli cell assessment; and (5) epigenetic studies of EDC effects on Sertoli cell programming.

## Conclusions

Incomplete male puberty represents a newly recognised clinical entity characterised by imperfect progression of testicular volume during puberty due to Sertoli cell dysfunction, in the presence of normal secondary sexual characteristics. The condition is clinically silent until the first spermiogram reveals azoospermia or severe oligozoospermia. A structured surveillance protocol—incorporating neonatal minipuberty screening, serial orchidometry, and targeted hormonal assessment—can identify affected boys years before irreversible spermatogenic failure. High-risk categories (cryptorchidism, offspring of diabetic or hypothyroid mothers, boys with significant EDC exposure) merit enhanced surveillance from birth. Widespread adoption of these strategies has the potential to transform the management of male infertility from reactive to preventive.
